# The Optimal Management of Inflammatory Bowel Disease in Patients with Cancer

**DOI:** 10.3390/jcm12062432

**Published:** 2023-03-22

**Authors:** Panu Wetwittayakhlang, Paraskevi Tselekouni, Reem Al-Jabri, Talat Bessissow, Peter L. Lakatos

**Affiliations:** 1 Division of Gastroenterology and Hepatology, McGill University Health Centre, Montreal, QC H3G 1A4, Canada; 2 Gastroenterology and Hepatology Unit, Division of Internal Medicine, Faculty of Medicine, Prince of Songkla University, Hat Yai 90110, Songkhla, Thailand; 3 Department of Internal Medicine and Oncology, Semmelweis University, 1085 Budapest, Hungary

**Keywords:** inflammatory bowel disease, ulcerative colitis, Crohn’s disease, cancer, risk, biologic, anti-tumor necrosis factors, thiopurine, vedolizumab, ustekinumab

## Abstract

Patients with inflammatory bowel disease (IBD) have an increased risk of cancer secondary to chronic inflammation and long-term use of immunosuppressive therapy. With the aging IBD population, the prevalence of cancer in IBD patients is increasing. As a result, there is increasing concern about the impact of IBD therapy on cancer risk and survival, as well as the effects of cancer therapies on the disease course of IBD. Managing IBD in patients with current or previous cancer is challenging since clinical guidelines are based mainly on expert consensus. Evidence is rare and mainly available from registries or observational studies. In contrast, excluding patients with previous/or active cancer from clinical trials and short-term follow-up can lead to an underestimation of the cancer or cancer recurrence risk of approved medications. The present narrative review aims to summarize the current evidence and provide practical guidance on the management of IBD patients with cancer.

## 1. Introduction

Inflammatory bowel disease (IBD) is a chronic, progressive, immune-mediated disorder of the gastrointestinal (GI) tract, including Crohn’s disease (CD) and ulcerative colitis (UC). IBD impacts patients’ quality of life and can result in irreversible long-term complications, including cancer [1,2]. Patients with IBD are at increased risk of cancer, both intestinal and extra-intestinal cancers, compared to the general population, approximately 1.1-fold for UC patients and 1.3-fold for CD patients [3]. IBD is associated with the development of cancer secondary to underlying chronic inflammation and long-term use of immunosuppressive or biological therapy [4,5].

Because the incidences of CD and UC are rapidly increasing globally, the prevalence of IBD is increasing as well, owing to the early age of disease onset, increased survival, and increased life expectancy in the ageing IBD population [6,7,8]. Patients with IBD and cancer are becoming more common in clinical practice. However, physicians are frequently confronted with the question of whether they should start, re-start, continue, or withdraw IBD medications.

Therapeutic strategies in IBD have been shifting from mere symptomatic control toward complete disease remission, as recommended in the current treat-to-target strategy in IBD [9,10]. Thus, this strategy may result in more aggressive therapy with immunomodulators and biological therapies in the earlier course of the disease and a prolonged duration of exposure to immunosuppressants [11]. Despite the benefit of tightly controlling intestinal inflammation, which reduces colorectal cancer risk in IBD patients, the risk of developing extra-intestinal cancers associated with the carcinogenic effect of long-standing immunosuppressive therapy is increasing consequently [12,13]. Moreover, advanced therapies in the treatment of cancer, such as immune checkpoint inhibitors, have become the new standard of care in several cancers [14]. There is increasing concern about the impact of IBD medications on the survival and progression of cancer, as well as the effects of cancer therapy on the disease course of IBD.

Management of IBD for patients with a cancer diagnosis is challenging. There are relatively few standard guidelines for the management of IBD in patients with cancer. Data from randomized controlled trials (RCTs) excluded patients with a known history of cancer and reported only a short-term risk. Thus, most evidence of cancer risk in IBD patients is based on data from observational or retrospective studies. Furthermore, there is a large variation in the risks of site-specific cancer in the different patient backgrounds and among IBD therapies.

The aim of this review is to discuss the current evidence on the impact of IBD therapies on the risk of the development or recurrence of cancer. Furthermore, we summarized the practical management of IBD in patients with active cancer and patients with a history of previous cancer.

## 2. Risk of Developing Cancer in Patients with IBD

A large Danish population-based cohort collected data from 30 years of follow-up, from 1978 through 2010, which showed that IBD patients had a slightly increased risk of cancer compared to the general population (CD; SIR: 1.3, 95% CI: 1.2–1.4; and UC; SIR: 1.1, 95% CI: 1.0–1.1). However, the risk of GI cancer in CD has decreased over time from 1.9-fold (1978–1987) to 0.9-fold (after 1987). Similarly, the risk of GI cancer in UC patients has decreased from 1.4 (95% CI: 1.0–1.8) to 1.1 (95% CI: 0.9–1.3) fold, suggesting the risk of GI cancers among IBD patients did not differ from the general population in the last two decades [3]. In the multicenter European Collaborative-IBD Study (1993–2009), the overall prevalence of intestinal and extraintestinal cancers was 9.1%, while the prevalence of CRC was 1.3% at 15 years after IBD diagnosis, and cancer prevalence was not different from that expected in the background population [15].

In a meta-analysis of eight population-based studies, IBD patients were not at increased risk of extra-intestinal cancer (EIC) compared to the background population (SIR 1.10; 95% CI: 0.96–1.27). However, site-specific analyses showed that patients with CD had an increased risk of upper GI tract (SIR 2.87), lung (SIR 1.82), urinary bladder (SIR 2.03), and squamous cell skin cancer (SIR 2.35). Whereas patients with UC had a significantly increased risk of hepatobiliary cancer (SIR 2.58) and leukemia (SIR 2.00) [16].

The mechanism of cancer pathogenesis in IBD can be divided into inflammation-related and immunosuppressive agent-related cancer [4,5]. Long-standing inflammation in IBD that can trigger tumor initiation and progression has been associated with certain cancer types, including colorectal carcinoma (CRC), small bowel adenocarcinoma, intestinal lymphoma, anal carcinoma, and cholangiocarcinoma (CCA). Thus, these cancers are potentially preventable with the use of immunosuppressive and biological therapy that can reduce inflammation, which results in reducing the risk of developing cancer. However, immunosuppressive and biologic therapy are associated with decreased immunosurveillance of cancers and facilitation of the action of oncogenic viruses. Secondary reactivation of latent Epstein–Barr virus (EBV) infection is linked to lymphoproliferative disorders. Young (35-year-old) men who are seronegative for EBV and are exposed to thiopurine therapy are at risk of developing fatal forms of primary EBV infection. Human papillomavirus (HPV) is linked to an increased risk of cervical and anal cancer [17]. For certain immunosuppressive medications, a direct oncogenic effect has been reported [17,18]. IBD therapy has been linked to an increased risk of extra-GI cancer, mainly skin cancer and hematologic malignancy. The classification of cancer in IBD patients stratified by the pathogenesis of cancer is shown in Table 1.

## 3. Inflammation-Related Cancer in Patients with IBD

### 3.1. Colorectal Carcinoma

An association between CRC and IBD has been clearly established. IBD patients are at increased risk for CRC except for patients without colonic inflammation and patients with limited disease to proctitis. An earlier meta-analysis reported that the cumulative risk for patients with UC was 2% at 10 years, 8% at 20 years, and 18% at 30 years [19].

In a meta-analysis of a population-based study in 2012 by Jess et al., patients with UC have a 2.4-fold increased risk of developing CRC compared to the general population, and 1.6% of UC patients were diagnosed with CRC over an average 14-year follow-up [20]. However, a more recent meta-analysis suggests that the risk of CRC decreased over the last several decades after the improvement of treatment for IBD and the implementation of CRC surveillance. The incidence rate decreased from 4.29/1000 patient-years (PY) in the studies published in the 1950s to 1.21/1000 PY in studies published in the 2010s [21].

The most important risk factor for IBD-associated CRC is extensive colitis and disease duration in both UC and CD [4,22]. In the CESAME study, a prospective observational cohort, the patients with long-standing extensive colitis had an increased CRC risk 7-fold compared to the general population (SIR 7.0; 95% CI: 4.4–10.5), whereas SIRs was 2.2 (95% CI: 1.5–3.0) for all IBD patients and 1.1 (95% CI: 0.6–1.8) in patients without long-standing extensive colitis [22]. Of note, the risk increases significantly 8–10 years after diagnosis or when dysplasia is detected on colonic biopsies [23]. In addition, co-existing primary sclerosing cholangitis (PSC) has a significantly increased CRC risk, particularly in patients with UC (HR: 2.43) [24].

A recent meta-analysis published in 2021 classified extensive colitis as the only strong predictor for developing CRC in patients with IBD, while the presence of low-grade dysplasia, strictures, PSC, post-inflammatory polyps, family history of CRC, and UC (versus CD) was considered moderate, and evidence for any dysplasia, colon segment resection, aneuploidy, male sex, and age was classified as weak predictors [25].

In contrast, 5-aminosalicylic acid (5-ASA) and thiopurine therapy are shown to be protective factors for CRC in IBD patients [26]. A meta-analysis of 2137 cases of IBD patients with colorectal neoplasia (of which 76% were cancers) revealed that exposure to 5-ASA was protective against CRC (RR 0.58, 95% CI: 0.45–0.74) and dysplasia (RR 0.54, 95% CI: 0.35–0.84). However, this association was significant only in UC but not in CD [26]. The protective effect of thiopurine has been shown in two recent meta-analyses [27,28] with a reduced risk of colorectal neoplasia (high-grade dysplasia and CRC) both in case-control (OR 0.49, 95% CI: 0.34–0.70) and cohort studies (RR 0.96, 95% CI: 0.94–0.98). However, this protective effect was not seen in IBD patients with extensive colitis or PSC [27]. Biological therapy has not been shown to have a protective effect in the reduction of CRC risk given the limited studies with long-term follow-up. A meta-analysis of 4 studies did not find a protective effect of anti-TNF therapy (OR 0.71, 95% CI: 0.14–3.67) [25].

### 3.2. Anal and Rectal Cancer

CD patients with an anal or perianal disease are at increased risk for anal cancer, particularly fistula-related cancer; however, it is a rare complication in CD [29]. Of note, fistula-related cancer typically develops in patients with longstanding perianal CD. These cancers include adenocarcinomas and squamous-cell carcinomas (SCC) that have no consistent relationship with HPV infection [4]. In recent data from the CESAME cohort, the incidence rates per 1000 PY were 0.38 for perianal fistula-related adenocarcinoma, 0.26 for anal squamous-cell carcinoma, and 0.77 for rectal cancer [30]. A multicenter study from the Netherlands reported cancer developed 25 years after CD diagnosis and 10 years after fistula diagnosis [31]. Anal SCC occurring in patients with long-standing anal lesions has been linked to chronic inflammation, HPV infection, and drug-induced immunosuppression [4,17].

### 3.3. Small Bowel Cancer

CD patients with small bowel involvement have an increased risk of small-bowel cancer (SBC), but the increased risk of SBC in UC is not clear [32]. However, the absolute risk of SBC in CD is very low, with a reported incidence of 0.24–0.3 per 1000 PY [29,33]. The most common locations are in the distal jejunum, which is the most frequently involved segment in CD. Histologically, small-bowel adenocarcinoma is the most common subtype, approximately 40% [34]. In a Danish population-based study, CD patients have an increased risk of SBA compared to the general population, with SIR 14.4 (95% CI: 8.78–22.20) [35]. In addition, CD patients with a stricturing disease, a fistulizing disease, prior surgical intestinal resections, and/or childhood onset have the highest risk of developing SBC [29,33,35,36].

### 3.4. Cholangiocarcinoma

The important risk factor for CCA in IBD patients is co-existing PSC, particularly in patients with UC [24,37]. The incidence of CCA in PSC patients without IBD or with CD is lower than in patients with UC (1.02 and 1.11 vs. 1.22 per 100 PY, respectively) [38]. Of note, CCA is diagnosed in up to 10% of PSC patients within the first 10 years following PSC diagnosis [39,40]. The risk of CCA in PSC increased with older age, male sex, and the presence of IBD [37,38].

## 4. Risk of IBD Therapy-Related Cancer

Although immunosuppressive and biological therapies are effective in controlling intestinal inflammation in IBD, they may cause tumor formation by altering tumor suppressor genes, impairing immune control of chronic infection, e.g., EBV or human papillomavirus (HPV), and reducing the immunosurveillance of cancer or dysplastic cells.

### 4.1. Thiopurine and Cancer Risk

Thiopurine use has been linked to an increased risk of certain specific cancers, particularly NMSC and lymphoma [41,42,43,44]. Whereas the overall risk of other solid cancers, including melanoma associated with thiopurine exposure, was not clear [45,46,47,48]. The increased risk of lymphoproliferative disorder was identified in the CESAME study, a large prospective observational cohort of 19,486 IBD patients during a mean follow-up of 35 months. The incidence rates of lymphoproliferative disorder were 0.90/1000 PY in those receiving it; 0.20/1000 PY in those who had discontinued it; and 0.26/1000 PY in those who were thiopurines naïve, *p* = 0.0054. The adjusted HR for lymphoproliferative disorders was 5.28 (95% CI: 2.01–13.90) in patients exposed to thiopurines compared with thiopurine naïve patients. Of note, the risk was higher in patients older than 50 years of age (2.58/1000 PY for 50–65 years and 5.41/1000 PY for older than 65 years in patients with continuing use) [41].

An earlier meta-analysis reported an approximate 4-fold increased risk of lymphoma in IBD patients treated with thiopurine (pooled RR 4.18, 95% CI: 2.07–7.51) [42]. A more recent meta-analysis of 18 studies published in 2014 confirmed that the overall SIR for lymphoma was 4.49 (95% CI: 2.81–7.17), ranging from 2.43 (95% CI: 1.50–3.92) in population-based studies to 9.16 (95% CI: 5.03–16.7) in referral studies. Of note, the risk became significant after 1 year of exposure (SIR 5.71, 95% CI: 3.22–10.1) and reverted to baseline after discontinuation (SIR: 1.42; 95% CI: 0.86–2.34). The absolute risk was highest in patients older than 50 years (1/377 cases per PY) [43]. Most thiopurine-associated lymphomas are B-cell lymphomas associated with EBV [49]. An analysis of the CESAME data showed a risk of 2.9/1000 PY for men under the age of 35 years at risk for fatal primary EBV infection, including polyclonal post-mononucleosis lymphoproliferation with thiopurine exposure [17].

Concerning the increased risk of NMSC-associated thiopurine therapy, the CESAME study showed the crude incidence rate of NMSC was 0.66/1000 PY in patients currently receiving thiopurines (HR 5.90, 95% CI: 2.13–16.4) and 0.38/1000 PY in patients who had previously received thiopurines (HR 3.9, 95% CI: 1.28–12.1). The increased risk of NMSC was observed even in patients younger than 50 years [44]. Consistently, a pooled analysis of 13 studies showed an excess risk of NMSC with thiopurines compared to non-thiopurine-treated patients (RR 1.88, 95% CI: 1.48–2.38) [50].

In contrast to NMSC, two meta-analyses reported no increased risk of melanoma in IBD patients exposed to thiopurine, with the most recent study reporting an RR of 1.22 (95% CI: 0.90–1.65) [50,51]. It remains unclear whether thiopurines are associated with a greater risk of cervical dysplasia/cancer in IBD patients [52,53]. Two population-based studies reported an increased risk of urinary tract cancer in IBD patients with thiopurine exposure [46,54], whereas another study did not find an increased risk [55]. For other site-specific solid cancers, including RCC, gastric cancer, breast cancer, and CCA, data from retrospective studies did not find an increased risk associated with thiopurine use in IBD patients [56,57,58,59].

### 4.2. Methotrexate and Cancer Risk

There is limited data on methotrexate (MTX) and cancer risk in patients with IBD. Only a large, case-control study reported an increased risk of NMSC in IBD patients exposed to MTX. Although the number of patients exposed to MTX alone was small (5 patients), this resulted in a very wide confidence interval (OR 8.55, 95% CI: 2.55–31.8). Moreover, this association was observed only in patients exposed to MTX for 1 year or less [60]. Data in RA patients showed a possible association between MTX use and NMSC [61,62]. However, other studies showed no association between MTX exposure and NMSC risk among IBD patients [63,64].

Multiple studies with relatively small numbers of MTX-exposed IBD patients and small numbers of incidences reported no increased risk of extra-colonic or site-specific cancer, including lymphoma, melanoma, NMSC, RCC, cervical cancer, and small-bowel carcinoma in IBD patients treated with MTX alone [48,56,63,64,65,66]. Nevertheless, several studies in patients with RA and psoriasis revealed that there was an excess risk of cancer among MTX-exposed patients compared to the general population (SIR 1.5, 95% CI: 1.2–1.9), with increased risk of melanoma (SIR 3.0, 95% CI: 1.2–6.2), non-Hodgkin’s lymphoma (SIR 5.1, 95% CI: 2.2–10.0), and lung cancer (SIR 2.9, 95% CI: 1.6–4.8). Thus, it is not possible to provide a precise cancer-specific risk of MTX in IBD patients.

### 4.3. Anti-Tumor Necrosis Factors (Anti-TNFs) and Cancer Risk

The current evidence shows that the overall risk of cancer in IBD patients treated with anti-TNFs is not increased. However, the risk of lymphoma and melanoma increased in patients receiving anti-TNF therapy. It is important to note that the accurate risk of cancer associated with anti-TNF is difficult to determine. First, there are pleiotropic effects of anti-TNFs and inflammatory pathways in IBD and tumorigenesis. Second, the majority of IBD patients who were treated with anti-TNFs had a severe or chronic continuous disease and had combination therapy with thiopurines [67]. Thus, disease severity and concomitant immunosuppressive agents could be potential confounding factors in estimating cancer risk in anti-TNFs.

Multiple meta-analyses reported no increased overall risk of cancer in IBD patients with anti-TNF therapy [68,69,70,71,72,73]. A recent systematic review by Muller et al. that included 28 observational cohort studies of 298,717 patients revealed that the overall risk of cancer in IBD patients treated with anti-TNF was comparable to that of anti-TNF naïve [68]. Similarly, a Danish nationwide study reported no increased risk of cancer among IBD patients exposed to anti-TNFs over a median follow-up of 3.7 years (RR 1.07, 95% CI: 0.85–1.36) [69]. In addition, there is no evidence of increased cancer risk associated with anti-TNF use in elderly IBD patients. Two meta-analyses also showed that the overall cancer risks in IBD patients older than 60 years of age were not increased by exposure to anti-TNFs (OR 0.5–0.9) [70,71].

Although the overall risk of cancer was not increased by anti-TNFs exposure, an increased risk of lymphoma in IBD patients receiving anti-TNFs has been reported in several studies [74,75,76,77]. In the Swiss IBD cohort of 3119 patients, increased lymphoma rates with anti-TNF were found in both CD (HR 3.26, 95% CI: 1.31–8.10) and UC patients (HR 25.25, 95% CI: 2.94–217.26) [74]. In 2020, a meta-analysis including 4 observational studies confirmed that anti-TNF therapy was associated with a higher rate of lymphoma than that in IBD patients unexposed to anti-TNFs with a pooled IRR of 1.52/1000 PY [75]. In line with an earlier meta-analysis of 26 studies, including 8905 patients, an increased risk for non-Hodgkin lymphoma (6.1/10,000 PY) was found, with SIR: 3.23 (95% CI: 1.5–6.9). However, 66% of these patients received combination therapy with thiopurine or MTX [76].

In contrast, a meta-analysis of RCTs included 74 RCTs of anti-TNFs; only 12 lymphoma cases were reported, with numbers too low to calculate HRs [78]. Similarly, two RCTs of adalimumab showed only 3 cases among 1010 UC patients diagnosed with lymphoma, all of them with thiopurine exposure, whereas no cases of lymphoma were reported in 1594 patients with CD [72,79]. Of note, the data from RCTs may represent the risk in RCT participants, who may be different from the general population because patients in the RCTs were selected and patients with pre-existing cancer risks or known cancer were excluded. Although, in a real-world prospective cohort of 5025 CD patients exposed to adalimumab, the PYRAMID registry observed that the lymphoma rate was 0.60/1000 PY, which was lower than the estimated background rate (0.84/1000 PY) [80]. Furthermore, data from the ENCORE cohort reassured that infliximab exposure was not associated with lymphoproliferative disorders or malignancy (HR 1.44, 95% CI: 0.86–2.42) [81].

Concerning the risk of skin cancer, anti-TNF therapy has a potentially increased risk of developing melanoma. However, the data were not solid [16,69,82,83]. A database study comprising 108,579 IBD patients, each matched with 4 controls without IBD, reported that anti-TNFs were associated with a significant increase in the risk of melanoma (OR 1.88; 95% CI: 1.08–3.29) [82]. In line with the results from nested case-control studies, anti-TNFs therapy increased the risk of melanoma (OR 1.88, 95% CI: 1.08–3.29) but not NMSC 1.14 (0.95–1.36) [82]. However, the studies could not control the confounders through prior or concomitant thiopurine exposure. Moreover, the increased melanoma risk has not been replicated in other studies [68,69]. A Danish population cohort revealed no association between anti-TNFs exposure and melanoma (RR 1.31, 95% CI: 0.63–2.74) [69]. Further, a recent meta-analysis that included 7901 IBD patients treated with anti-TNFs did not find an increased risk of anti-TNF exposure compared with non-biologic exposure (RR 1.20, 95% CI: 0.60–2.40) [83].

Regarding the risk of NMSC, in a systematic review that included 28 studies, 692 cancers were diagnosed in IBD patients treated with anti-TNFs, accounting for an overall occurrence of 1.0%. The most frequent malignancies were NMSC (123/692; 17.8%) and were reported at the same rates as expected in the general non-IBD population [68].

### 4.4. Combined Anti-TNF and Thiopurine Therapy and Cancer Risk

The current evidence shows that there is no additional increased risk of solid-organ or skin cancer (melanoma and NMSC) in IBD patients treated with combination therapy (anti-TNFs and thiopurine or MTX) compared to the risk in patients treated with anti-TNFs or thiopurine monotherapy [69,73,84]. However, the risk of lymphoma associated with combined anti-TNF and thiopurine therapy is significantly higher than that of thiopurine or anti-TNF monotherapy. In a French nationwide cohort, the incidence rates were 0.54, 0.41, and 0.95 per 1000 PY in IBD patients exposed to thiopurine monotherapy, anti-TNF monotherapy, and combination therapy, respectively. The risk of lymphoma was significantly higher among patients exposed to combination therapy (HR 6.11) than in those exposed to thiopurine monotherapy (HR: 2.60) or anti-TNF monotherapy (HR: 2.41) compared to unexposed patients [77]. Additionally, in a meta-analysis of 4 observational studies, the risk of lymphoma associated with combination therapy was higher than that with thiopurines or anti-TNFs alone (pooled IRR vs. thiopurines: 1.70; 95% CI: 1.03–2.81; pooled IRR vs. anti-TNFs monotherapy: 2.49; 95% CI: 1.39–4.47) [75].

In addition, despite a very high incidence rate, hepatosplenic T-cell lymphoma (HSTCL) has been reported in IBD patients with combination therapy. However, the risk was comparable with thiopurine monotherapy [76]. Most patients with HSTCL were exposed to thiopurine for at least 2 years and were young men (<35 years old) with CD [85,86].

### 4.5. Vedolizumab and Cancer Risk

The 4-year follow-up data from the global post-marketing database, which included 32,752 IBD patients treated with vedolizumab (VDZ), showed that VDZ exposure did not increase the overall risk of cancer. The incidence of cancer was reported in less than 1% of UC patients treated with VDZ. The most common cancer was GI cancer. However, the data were limited by the lack of a comparator group [87]. The GEMINI long-term safety study also reported no significant increase in the risk of cancer with VDZ exposure compared to controlled-IBD patients using age- and sex-specific rates of cancer. Thus, the gut-selective α4β7 integrin antibody, VDZ, appeared to have a favorable safety profile in terms of cancer risk [88,89]. However, long-term data are scarce and limited by the number of studies.

### 4.6. Ustekinumab and Cancer Risk

There is no increased risk of cancer observed in IBD patients treated with ustekinumab (UST). Post hoc analysis from the IM-UNITI trial up to 5 years of follow-up revealed no increased risk of cancer in CD patients treated with UST compared to non-UST exposure. The rates of cancer were 1.70/100 PY in the placebo group and 1.48/100 PY in the UST group [90]. There is no evidence of a significantly increased overall cancer risk in UC patients treated with UST (IR; UST: 0.72 vs. placebo: 0.66) [91]. Similar to the results from real-world registry observational studies, the incidence of cancer in IBD patients treated with UST was rare [92,93,94]. In addition, the PSOLAR registry revealed the rates of cancer (excluding NMSC) in psoriasis patients with long-term UST exposures were comparable with those expected in the general population [95]. However, data regarding cancer in UST were limited due to the lack of long-term follow-up, and most of the data were derived from RCTs.

### 4.7. Small Molecules Therapy (JAK Inhibitors) and Cancer Risk

Data regarding the cancer risk of JAK inhibitors in IBD patients are limited. Accordingly, most evidence is extrapolated from other immune-mediated diseases. In a meta-analysis of 82 RCTs comprising over 66,000 patients with immune-mediated diseases who were exposed to JAK inhibitors, the incidence rate of NMSC was higher in JAK inhibitor exposure compared to that in the comparators (0.51/100 PY vs. 0.27/100 PY), but the relative risk of NMSC associated with JAK inhibitors compared with placebo or an active comparator was not significantly increased (RR: 1.21, 95% CI: 0.19–7.65) [96]. While larger data on malignancy risk associated with JAK inhibitors reported from patients with RA is controversial. A pooled analysis of phase 2–3 studies of tofacitinib showed SIRs for all cancers (excluding NMSC) and selected cancers (lung, breast, lymphoma, NMSC) were within the expected range for patients with moderate-to-severe RA [97]. In contrast, a recent large RCT comparing the safety of tofacitinib and anti-TNF in patients with RA > 50 years of age and with at least one additional cardiovascular risk factor reported a higher incidence of overall cancer (excluding NMSC) with tofacitinib than with anti-TNFs therapy (HR 1.48; 95% CI: 1.04–2.09), particularly lung cancer and lymphoma [98].

**Figure 1:**
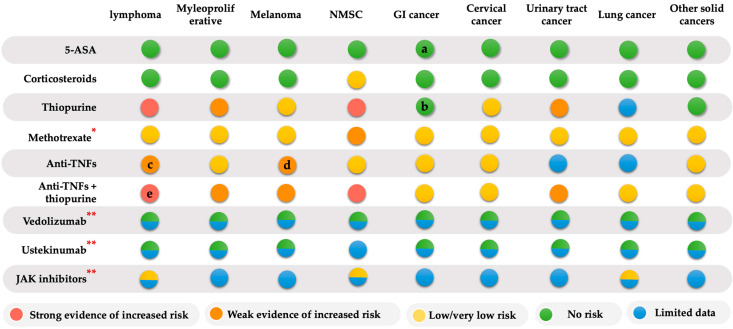
The risk of type-specific cancer associated with IBD therapies Note: a: 5-ASA has a protective effect for CRC; b: thiopurine has a protective effect for CRC and high-grade dysplasia; c: the increased risk was reported in meta-analyses and observational studies but not replicated in several studies, including RCTs, meta-analyses, and registry cohorts; d: the increased risk was reported in large population-based and case-control studies, but meta-analyses did not find an increased risk; e: insufficient data on the risk of lymphoma in IBD patients exposed to anti-TNF in combination with methotrexate. * Data on the risk of cancer in MTX alone were relatively limited, based on a small number of MTX-exposed patients and small numbers of cancer events. ** For vedolizumab, ustekinumab, and JAK inhibitors, long-term data are limited. No increased risk was reported with VDZ and UST exposures (excluding NMSC in UST). For JAK inhibitors, one safety RCT reported an increased risk of overall cancer, particularly lymphoma and lung cancer.

In the present review, we classified the IBD therapies associated with cancer risk based on the level of evidence using the Oxford methodology, [99] divided into: (1) Strong evidence of increased risk (evidence level, E 1, 2); much data were derived from meta-analyses, RCTs, or prospective comparative studies that consistently reported a significantly increased risk of cancer. (2) Weak evidence of increased cancer risk (EL 3, 4); data from retrospective or case-control studies on the increased cancer risk were not replicated among the studies. (3) Low or very low evidence of increased risk of cancer (EL 5); increased risk of cancer was reported from case reports or expert opinions. (4) No risk; data does not show the increased cancer risk in the available studies. (5) No or limited data; limited or lacking data on cancer risk. The risk of type-specific cancer associated with IBD therapies is summarized in Figure 1.

In summary, IBD therapies are not associated with an increased overall risk of cancer. However, they are associated with an increased risk of certain site-specific cancers. Thiopurine exposure increases the risk of lymphoma and NMSC. Particularly, young (<35-year-old) men receiving thiopurine treatment who are EBV-seronegative are at an increased risk of fatal primary EBV infection. Thus, physicians should consider and discuss the risk with patients before initiation of treatment. The risks of lymphoma/HSTCL were also observed to be significantly greater when patients received a combination therapy of anti-TNF and thiopurine. Whereas anti-TNF monotherapy potentially increased the risk of lymphoma and melanoma. For the new biologics (VDZ and UST), current evidence has not shown an increased overall cancer risk. However, there is a lack of long-term and large studies to draw a solid conclusion. JAK inhibitors may be associated with an increased risk of cancer, particularly lymphoma and lung cancer. There is very limited data on the cancer risk in patients treated with dual-targeted therapy, and the safety data of combined biological therapy were reported only in case series with a short follow-up period. The potential increased further risk of developing cancer should be discussed with the patient at the initiation of immunosuppressive and/or biological therapy.

## 5. Management of IBD Therapy in Patients with a History of Previous Cancer

IBD patients with a history of previous cancer have an overall increased risk of 1.9-fold of developing any (new or recurrent) cancer compared to IBD patients without a previous cancer, with an overall cancer incidence rate of 21.1/1000 PY in IBD patients with a prior cancer [45]. According to the relatively small numbers of IBD patients with previous cancer, most data are drawn from patients with post-organ transplantation or other immune-mediated inflammatory diseases to estimate the site-specific cancer risks. In general, myeloma, skin cancer, and lung and GI cancer are considered to be at higher risk of recurrence. Lymphoma, testicular, and cervical cancer were at lower risk of recurrence [100,101] (Table 2).

In the CESAME study analyzing data from 17,047 IBD patients with previous cancer, there was no significant increase in the risk of overall (new or recurrent cancers) in the IBD patients exposed to immunosuppressants, including thiopurines, MTX, and anti-TNF (new cancer; 23.1 vs. 13.2/1000 PY, and recurrent cancer; 3.9 vs. 6.0/1000 PY for exposure and non-exposure to immunosuppressants, respectively) [45]. In a retrospective study assessing the risk of recurrence in patients with breast cancer, there was no significantly increased risk of cancer recurrence with the use of MTX (HR 1.07, 95% CI: 0.67–1.69), anti-TNFs (HR 1.13, 95% CI: 0.65–1.97), or thiopurines (HR 2.10, 95% CI: 0.62–7.14) [58]. Furthermore, a meta-analysis of 16 studies, including 11,702 patients with an immune-mediated inflammatory disease and a history of previous cancer, confirmed that the rate of recurrent cancer was not higher in patients receiving immunomodulators than that in patients without an immunomodulator or anti-TNF (anti-TNF: 33.8/1000 PY vs. immunomodulator: 36.2/1000 PY vs. no immunosuppression: 37.5/1000 PY). However, the risk was numerically higher among patients with combination therapy of anti-TNFs and immunomodulators (54.5/1000 PY). The rates of new or recurrent cancer were also similar in patients receiving thiopurine or MTX. These findings were consistent in a subgroup analysis of the 3706 patients with IBD. However, in the sub-group of patients with previous skin cancer, the risk of new or recurrent cancers was greater in patients exposed to immunomodulators than in those exposed to non-immunosuppressants (71.6/1000 PY vs. 50.8/1000 PY, *p* = 0.035) [102]. In a meta-analysis of 9 observational studies, the pooled IRR of new or recurrent cancer among patients with a history of cancer exposed to anti-TNFs therapy was not significantly different compared to control therapies, with an IRR of 0.90 (95% CI: 0.59–1.37) for immune-mediated inflammatory disease and an IRR of 1.06 (95% CI: 0.59–1.37) for IBD patients [103].

Regarding data concerning new biologic therapies, a recent multicenter retrospective study included 538 IBD patients and compared the risks of incident cancer in patients with a history of non-GI cancer and receiving thiopurines (27%), anti-TNF (21%), or VDZ (9%). The crude cancer incidence rates per 1000 PY were 47.0 for patients receiving no immunomodulator, 36.6 in the anti-TNFs cohort, and 33.6 in the VDZ cohort, *p* = 0.23. Incident-cancer-free survival rates were not different between patients receiving anti-TNF and those receiving VDZ, *p* = 0.56. After adjustment, incidence rates were not different between patients receiving no immunomodulator, anti-TNF, or VDZ [104].

**Figure 2:**
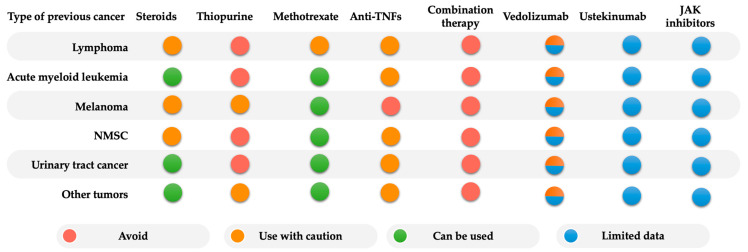
The risk of type-specific cancer associated with IBD therapies Management of IBD therapy in patients with a history of previous cancer (adapted from ECCO guideline 2015 [106]). Abbreviations: anti-TNF—anti-tumor necrosis factors; NMSC—non-melanoma skin cancer; JAK inhibitors—Janus kinase inhibitors.

The most recent study, published in 2022 by Vedamurthy et al., analyzed 463 IBD patients. A total of 96 patients were exposed to VDZ, 184 were exposed to anti-TNF, and 183 had no immunosuppressive therapy after a prior cancer diagnosis. Among VDZ-treated patients, 18 patients developed new or recurrent cancer, corresponding to a rate of 22/1000 PY after a cancer diagnosis. There was no increase in the risk of new or recurrent cancer with VDZ (HR 1.38, 95% CI: 0.38–1.36) or anti-TNF therapy (HR 1.03, 95% CI: 0.65–1.64) when compared to non-immunosuppressive therapy. The study suggested that VDZ can be considered in IBD patients with a prior diagnosis of cancer [105]. Of note, there are still limited data on the effect of UST and JAK inhibitors on the risk of cancer recurrence in IBD patients with previous cancer. The risk for management of IBD in patients with previous cancer is shown in Figure 2.

In summary, based on available evidence, there is no additional increased risk of new or recurrent cancer with thiopurine, MTX, or biologic therapy, including anti-TNF and VDZ, in IBD patients with a history of previous cancer beyond the known risk in general IBD patients (without previous cancer). However, it is important to note that most data are from patients starting thiopurine or anti-TNF more than 5 years after cancer resolution and in patients with a low risk of cancer recurrence [23,106].

A minimum interval of 2 years for a drug holiday is suggested by the ECCO statement before starting or resuming immunosuppressive or biological therapy in cancers with a low-intermediate risk of recurrence [106], given that 20% of cancer recurrence usually occurs within the first 2 years [101]. Of note, thiopurines should only be considered if no other treatment options are available and after the minimum of 5 years following cancer resolution in patients at high risk of cancer recurrence. Anti-TNFs can be started or continued as monotherapy, except in the setting of melanoma as a high-risk recurrence cancer [106]. The combination therapy of anti-TNFs with thiopurine should be avoided in IBD patients with prior cancer. Even though it may be unnecessary to conservatively follow the 2-year drug holiday approach, especially when considering the risk of not treating IBD effectively [107]. Treatment decisions can be individualized according to the risk of cancer recurrence, IBD disease activity, and patient risk preferences.

**Figure 3:**
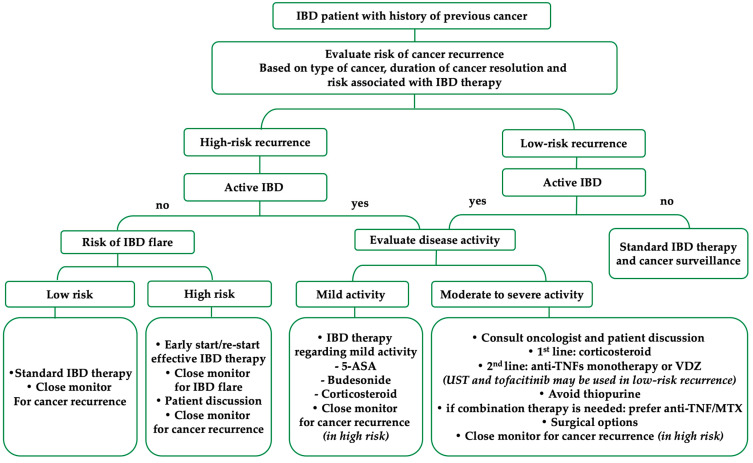
The practical treatment algorithm for the management of IBD in patients with a previous history of cancer. Abbreviations: IBD—inflammatory bowel disease; anti-TNF—anti-tumor necrosis factors; MTX—methotrexate; VDZ—vedolizumab; UST—ustekinumab; 5-ASA—5-amino salicylic acid.

Although there are limited data on the long-term risk of cancer recurrence with new biologics and JAK inhibitors. In patients with active and severe IBD, VDZ can be used in selected cases with caution after careful risk consideration. However, the cancer risk associated with UST and JAK inhibitors is limited in patients with previous cancer. The practical treatment algorithm for the management of IBD in patients with a history of previous cancer is shown in Figure 3.

## 6. Management of IBD Therapy in Patients with Current or Active Cancer

The management of IBD patients with active cancer remains challenging, as IBD therapies may impact the cancer’s course and survival. On the other hand, extensive and metastatic cancers, as well as the treatment of cancer, may worsen the course of IBD [108]. Although a prospective study reported that the diagnosis of cancer was not associated with significant changes in IBD activity, it led to some changes in the IBD therapies, with a lesser use of thiopurines (pre- and post-cancer diagnosis: 25% vs. 19%, *p* < 0.001) and an increased need for intestinal surgery (2.5% vs. 4.0%, *p* = 0.05) [109]. Moreover, active IBD may complicate the choices of therapies and potential outcomes of cancer [67]. The goal of IBD treatment is to control the disease activity of IBD (mainly clinical remission) and prevent IBD flare-ups during the course of cancer treatment in order to allow the patient to complete the cancer treatment without complications or the need for surgery.

### 6.1. Management of IBD Therapies in Patients with Active Cancer

For IBD patients with a current diagnosis or active cancer, thiopurines should be withdrawn [23,101,106]. Given a potential risk related to mutations in tumor suppressor genes, T-cell suppression, and bone marrow suppression [110]. Thiopurines should be withheld during the treatment of cancer or until the cancer is controlled. For patients with cancers or pre-neoplastic lesions that are considered to be at low risk of recurrence and that have been successfully removed endoscopically or surgically, such as non-aggressive basal cell carcinoma, cervical dysplasia, or sporadic colonic polyps, thiopurines can be continued with closed monitoring for cancer surveillance [23].

In a retrospective cohort of 14 IBD patients diagnosed with lymphoma, 50% of patients were treated with thiopurine. The survival rate was similar to the expected survival for both thiopurine-treated and untreated patients. However, statistical analysis was limited by the small sample size and heterogeneity of the patients studied [111]. There are insufficient data on whether MTX has a negative impact on cancer progression or prognosis [23].

Anti-TNF can be used in IBD patients with current cancer [23], according to the data from the Swedish observational cohort, which included 78,483 patients with RA treated with biologics (98% were anti-TNF). In the patients with a diagnosis of cancer, anti-TNF-exposed patients were matched (for cancer site, sex, age, and year of cancer diagnosis) with the non-anti-TNF-exposed patients. The death rate following cancer diagnosis was 113 deaths among 302 patients with anti-TNF therapy vs. 256 deaths among 586 patients in the non-anti-TNF exposure group. The relative risk of death following cancer associated with anti-TNF exposure was not significant (RR 1.1, 95% CI: 0.8–1.6). However, the study provided only an association between anti-TNF therapy and cancer outcome, not the effects of continuing anti-TNF therapy after the diagnosis of cancer. Of note, most patients discontinued anti-TNF therapy at cancer diagnosis [112]. Whereas there are insufficient data regarding the safety of VDZ, UST, or JAK inhibitors in IBD patients with active cancer. Only one study showed no increase in the risk of new or recurrent cancer with VDZ and anti-TNFs therapy compared to non-biological therapy, but the study analyzed patients with previous cancer, not active cancer patients [105].

### 6.2. Management of Chemotherapy and Radiation Therapy in IBD Patients

There are pros and cons of chemotherapy in IBD patients. Several studies reported the benefit of chemotherapy on the remission of IBD [108,113]. Whilst some proportions of patients have IBD flare following chemotherapy or adjuvant hormonal therapy. In a retrospective study of 84 IBD patients who received cancer treatment, among patients with active IBD at cancer diagnosis, 66.7% (n = 10/15) achieved remission during cancer treatment [108].

On the other hand, in the IBD patients in clinical remission at cancer diagnosis, 17.4% (n = 12/69) developed a flare of IBD after chemotherapy. At 5-year follow-up, 90% of those patients who received cytotoxic chemotherapy remained in clinical remission compared with 64% of those who received only hormone therapy or the combination of cytotoxic chemotherapy and adjuvant hormone therapy, *p* = 0.02 [108]. Another study of 41 IBD patients showed that the rates of IBD flare after chemotherapy were lower compared to the rates before starting chemotherapy (0.3/5 years vs. 1.4/5 years, *p* < 0.01), and the need for 5-ASA (47% vs. 71%, *p* < 0.01) and corticosteroids (9% vs. 32%, *p* = 0.02) were also decreased after chemotherapy [113]. A most recent systematic review and meta-analysis published in 2023 showed that the overall occurrence of IBD flares following cancer treatment was 30% (95% CI: 23–37%). IBD flares resulted in the utilization of systemic steroids and biological therapies among 25% and 10% of patients, respectively, and in the discontinuation of cancer treatment among 14% of patients. Most studies generally reported that flares were manageable [114].

There are some concerns about the toxicity of radiotherapy for cancer in patients with IBD. Thus, many oncologists prefer to avoid pelvic radiotherapy in cases of IBD [115]. In a retrospective study of 100 IBD patients with prostate cancer, 47% received radiation therapy. IBD flares were 2-fold higher for radiation-treated patients within 6 months (10.6% vs. 5.7%), and 6–12 months (4.3% vs. 1.9%) after a cancer diagnosis. Radiation treatment (OD 4.82, 95% CI: 1.15–20.26) was a predictor of IBD flares. However, there were no differences in IBD-related hospitalizations or surgeries [116]. Additionally, another study confirmed that the 5-year survival of rectal cancers in patients with IBD treated with pelvic radiation was similar to that of those with no prior IBD and that there was no increase in gastrointestinal toxicity [117]. In addition, a systematic review of 19 studies comprising 497 patients (GI cancer: 55% and prostate cancer: 40%) revealed that radiation therapy appears to be safe with an acceptable toxicity profile in IBD patients.

Therefore, regarding the priority given to cancer treatment, although patients should be counseled about the increased risks of an IBD flare, avoidance of chemotherapy or radiotherapy in IBD patients is not necessary.

### 6.3. Management of Immune Checkpoint-Inhibitor (ICIs) Associated with IBD

Immune checkpoint inhibitors (ICIs) targeting cytotoxic T-lymphocyte-associated protein-4 (CTLA-4) and programmed death-1/ligand (PD-1/PD-L1) lead to immune-related adverse events (irAEs), of which GI irAEs are among the most frequent and usually severe [14]. In a retrospective study of cancer patients treated with ICIs, 4 of 21 IBD patients (19%) had flared a median of 7 weeks (range 4–40) after starting ICIs [118]. The risk of GI toxicity following ICIs was increased 3-fold in patients with IBD compared to patients without IBD (RR 3.62, 95% CI: 2.57–5.09) [114].

More frequent irAEs are ICIs-induced enterocolitis, which shares similar phenotypical, endoscopic, and histological features with IBD [119]. It is important to investigate and exclude the common causes of diarrhea in immunocompromised patients, particularly infectious colitis (e.g., clostridium difficile and cytomegalovirus infection). In patients with ICI-induced enterocolitis, oral corticosteroids (prednisolone 0.5–1 mg/kg/day) should be added in case of inadequate response to conservative treatment [120]. Patients with severe toxicity, defined as an increase of ≥7 stools per day over baseline, needed hospitalization, severe or persistent abdominal pain, and/or the presence of life-threatening conditions, should discontinue ICIs and receive methylprednisolone (1 mg/kg/day).

In patients who fail to respond to intravenous corticosteroids, infliximab (IFX, 5 mg/kg) is indicated. A single dose of IFX is often sufficient to improve symptoms, although a second infusion 2 weeks later may be needed in some cases [119,121,122]. In an observational study of 39 patients with anti-CTLA-4-induced enterocolitis, 37% of patients treated with steroids achieved clinical remission, 12 patients required IFX, and 83% of those responded [119]. There was no difference in cancer outcome in the patients treated with a short course of IFX treatment, implying that IFX can be used in the setting of active cancer with co-existing ICI-induced enterocolitis [14,119]. A recent case series of 7 patients who received VDZ revealed that VDZ is effective and well-tolerated for steroid-dependent or partially refractory ICIs-induced enterocolitis, with clinical remission and fecal calprotectin normalization within 8 weeks [123]. In addition, the efficacy of tofacitinib (dose 5–10 mg/kg 2–3 times a day) has been recently reported in case series for patients with steroid-dependent or biologics-refractory ICIs-induced enterocolitis [124].

In summary, based on the current evidence and treatment guidelines [23,67,101,106], the best approach for IBD treatment in patients with active cancer should be discussed case-by-case according to the type, stages, and treatment strategy of cancer and the disease activity of IBD. Therefore, a multidisciplinary approach to decision-making involving gastroenterologists and oncologists to provide careful patient counseling should be implemented. 5-ASA and corticosteroids are considered safe and can be used as the first line of treatment in patients with clinically active IBD. Anti-TNFs can be used as second-line therapy in patients who are corticosteroid non-responders. Importantly, thiopurine should be discontinued until the cancer is controlled. Anti-TNFs therapy can be continued, except in patients with melanoma. Despite the limited data, VDZ has been reported to have efficacy and safety in IBD patients with active cancer and in the setting of ICI-induced enterocolitis.

**Figure 4:**
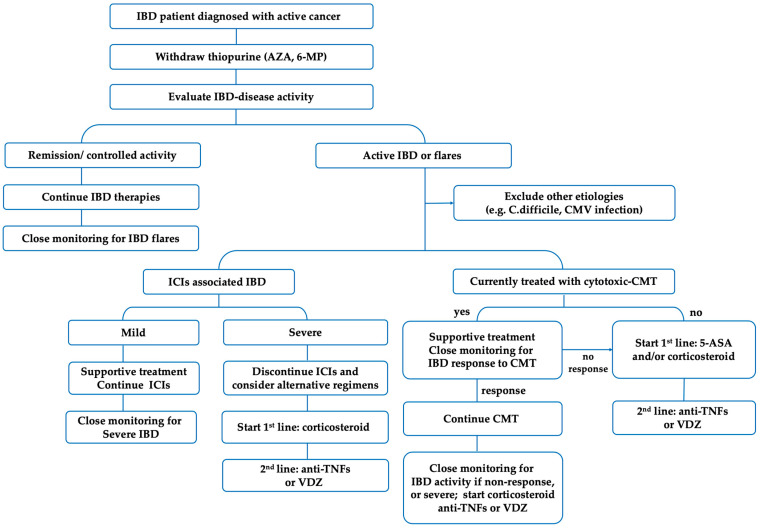
The practical treatment algorithm for the management of IBD in patients with active cancer. Abbreviations: IBD—inflammatory bowel disease; AZA—azathioprine; 6-MP—Mercaptopurine; CMT—chemotherapy; ICIs—immune checkpoint inhibitors; anti-TNF—anti-tumor necrosis factors; VDZ—vedolizumab.

Chemotherapy and radiation therapy may increase the risk of IBD flares. However, the flares are usually manageable with medical treatment and should not preclude appropriate cancer treatments. IBD patients may have the potential benefit of cytotoxic chemotherapy for inducing or maintaining IBD remission. Therefore, given the priority of increasing cancer survival, it is reasonable to continue cancer therapy under close monitoring for IBD flares. There is insufficient data on UST and tofacitinib in the treatment of IBD in patients with active cancer. The practical treatment algorithm for the management of IBD in patients with active cancer is shown in Figure 4.

## 7. Conclusions

**Figure 5:**
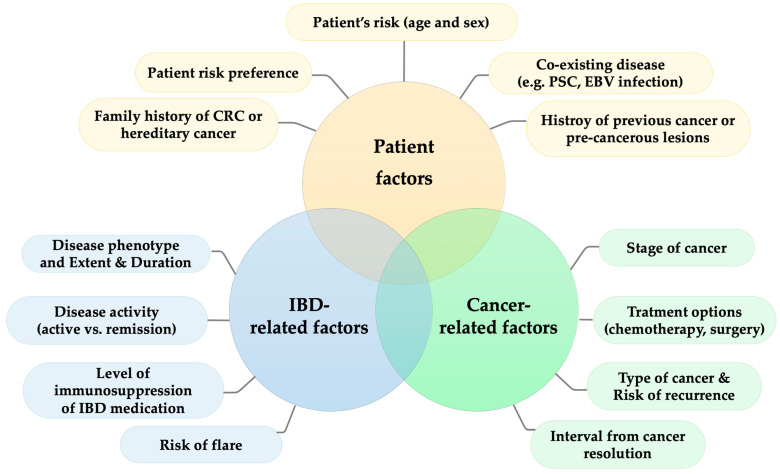
Conceptual framework of decision-making for treatment of IBD in patients with cancer.

It is essential to balance the benefit of IBD medications and cancer risk in IBD management before making a treatment decision, particularly in the setting of active IBD in patients with active cancer or a history of previous cancer. A case-by-case discussion involving gastroenterologists, oncologists, surgeons, and patients is needed to optimize the best treatment outcomes. Physicians should also be aware that even when treating with the same medication, the risks of cancer in an individual are different based on the different patient background and different types of cancer. The personalized decision is warranted on the basis of patient risk of cancer and patient preference. IBD-related risk, regarding disease severity/activity, IBD medication, and the risk of an IBD flare; and cancer-related risk, including the risk of cancer recurrence and the interval between cancer resolution, should be taken into account, as summarized in Figure 5.

## Figures and Tables

**Table 1:** Classification of cancer in IBD patients.

Inflammation-Related Cancer	IBD Therapy-Related Cancer
Colorectal cancer	Melanoma
Small intestinal cancer	Non-melanoma skin cancer
Intestinal lymphoma	Lymphoproliferative, hematological malignancy
Anal carcinoma	Cervical cancer
Cholangiocarcinoma	Urinary tract cancer

**Table 2:** Classification of cancer according to the risk of recurrence.

Low Risk (<10%)	Intermediate Risk (11–25%)	High Risk (>25%)
Lymphoma (HL and NHL)	Uterine body	Myeloma
Thyroid	Gastrointestinal cancer, colon	Skin cancer (Melanoma and NMSC)
Uterine and cervix	Prostate	Symptomatic renal carcinoma
Testicle	Breast	Bladder
Incidental asymptomatic renal tumor	Lung	Sarcoma

Abbreviations: HL—Hodgkin’s disease; NHL—non-Hodgkin lymphoma; NMSC—non-melanoma skin cancer.

## Data Availability

Not applicable.
